# Transcriptomic Analysis Reveals That Retinal Neuromodulation Is a Relevant Mechanism in the Neuroprotective Effect of Sitagliptin in an Experimental Model of Diabetic Retinopathy

**DOI:** 10.3390/ijms24010571

**Published:** 2022-12-29

**Authors:** Hugo Ramos, Patricia Bogdanov, Rafael Simó, Anna Deàs-Just, Cristina Hernández

**Affiliations:** 1 Diabetes and Metabolism Research Unit, Vall d’Hebron Research Institute, Universitat Autònoma de Barcelona, 08035 Barcelona, Spain; 2 Centro de Investigación Biomédica en Red de Diabetes y Enfermedades Metabólicas Asociadas (CIBERDEM), Instituto de Salud Carlos III (ICSIII), 28029 Madrid, Spain

**Keywords:** dipeptidyl peptidase-4 inhibitors, sitagliptin, differentially expressed RNAs, synaptic signal transmission, diabetic retinopathy

## Abstract

Synaptic dysfunction and neuronal damage have been extensively associated with diabetic retinopathy (DR). Our group evidenced that chronic hyperglycemia reduces the retinal expression of presynaptic proteins, which are crucial for proper synaptic function. The aim of the study was to explore the effect of topically administered sitagliptin, an inhibitor of the enzyme dipeptidyl peptidase-4, on the retinal expression patterns of an experimental model of DR. Transcriptome analysis was performed, comparing the retinas of 10 diabetic (db/db) mice randomly treated with sitagliptin eye drops (10 mg/mL) twice daily and the retinas of 10 additional db/db mice that received vehicle eye drops. Ten non-diabetic mice (db/+) were used as a control group. The Gene Ontology (GO) and Reactome databases were used to perform the gene set enrichment analysis (GSEA) in order to explore the most enriched biological pathways among the groups. The most differentiated genes of these pathways were validated through quantitative RT-PCR. Transcriptome analysis revealed that sitagliptin eye drops have a significant effect on retinal expression patterns and that neurotransmission is the most enriched biological process. Our study evidenced enriched pathways that contain genes involved in membrane trafficking, transmission across chemical synapses, vesicle-mediated transport, neurotransmitter receptors and postsynaptic signal transmission with negative regulation of signaling as a consequence of neuroprotector treatment with sitagliptin. This improves the modulation of the macromolecule biosynthetic process with positive regulation of cell communication, which provides beneficial effects for the neuronal metabolism. This study suggests that topical administration of sitagliptin ameliorates the abnormalities on presynaptic and postsynaptic signal transmission during experimental DR and that this improvement is one of the main mechanisms behind the previously demonstrated beneficial effects.

## 1. Introduction

The ceaseless progression of diabetes mellitus is turning diabetic retinopathy (DR) into the major source of preventable blindness among working-aged adults worldwide [1]. Due to the lack of symptoms during the early stages of DR, current diagnostic and therapeutic strategies address the advanced stages of the disease [2]. Furthermore, current treatments are expensive, invasive and are associated with side effects. Targeting classic risk factors of diabetes (mainly hyperglycemia and hypertension) is the only strategy against the early stages, giving rise to an unmet medical necessity [3].

The impairment of the neurovascular unit (NVU) of the retina, a functional coupling that integrates vascular flow with metabolic activity and that maintains the integrity of the inner blood–retinal barrier (iBRB), is an early event in the pathogenesis of DR [4,5]. Neurodegeneration, glial activation and early microvascular abnormalities are its main hallmarks [5].

New experimental approaches targeting the early neurodegenerative processes that occur in diabetic retinas are currently being explored [6]. The incretin glucagon-like peptide-1 (GLP-1) is a hormone that has been related to neuroprotection in the central nervous system (CNS) and, consequently, has been postulated as a potential candidate for the treatment of DR due to the direct embryological relationship between the CNS and the retina [7]. Recently, GLP-1, its receptor, the GLP-1 receptor (GLP-1R), mainly responsible for its degradation, and the enzyme dipeptidyl peptidase-4 (DPP-4) were detected in murine and human retinas [8,9]. The low retinal levels of GLP-1 reported in diabetic patients suggest that its replacement treatment could have a neuroprotective role against early DR [5].

Topical administration (eye drops) of GLP-1R agonists (GLP-1RAs) and topical administration of DPP-4 inhibitors (DPP-4i) are two different strategies that seek a common neuroprotective effect: GLP-1R activation. Their effectiveness has been already demonstrated in an experimental model of DR, preventing glial activation, cell death, glutamate excitotoxicity, vascular leakage and electroretinogram (ERG) abnormalities [8,9]. Nevertheless, the lower price, the higher stability and the emergence of new evidence that proves the neuroprotective role of other DPP-4 substrates place DPP-4i as a more attractive therapeutic strategy against the early stages of DR. The complexity of DPP-4i, derived from the multifunctional activity of the inhibited enzyme, and the need for enough preclinical evidence invite elucidation of the mechanisms behind their neuroprotective effects [10]. For this purpose, a transcriptome analysis was performed to study the effect of one DPP-4i, sitagliptin, in an experimental model of DR.

## 2. Results

### 2.1. Multiple Comparisons between Transcriptomes Revealed a Clear Effect of Both the Diabetic Condition and Sitagliptin Treatment

The largest number of differentially expressed genes (DEGs) was found in the comparison between sitagliptin-treated db/db mice and control db/+ mice (108 up-regulated and 33 down-regulated genes with an adjusted *p*-value (Adj.P. Val) of less than 0.05). The effect of the diabetic condition was represented by the comparative analysis between the vehicle-treated db/db and the control db/+ mice, which resulted in 19 DEGs (10 up-regulated and 9 down-regulated genes). At the same level of significance, transcriptomic analysis revealed a clear effect of sitagliptin eye drops in diabetic mice, which can be seen in the comparison between the sitagliptin-treated db/db mice and the vehicle-treated db/db mice, where 20 DEGs (17 up-regulated and 3 down-regulated genes) were obtained (Table 1).

### 2.2. Db/db Mice Topically Treated with Sitagliptin Showed Different Expression Patterns in the Retina Compared to Those Treated with Vehicle

**Figure 1:**
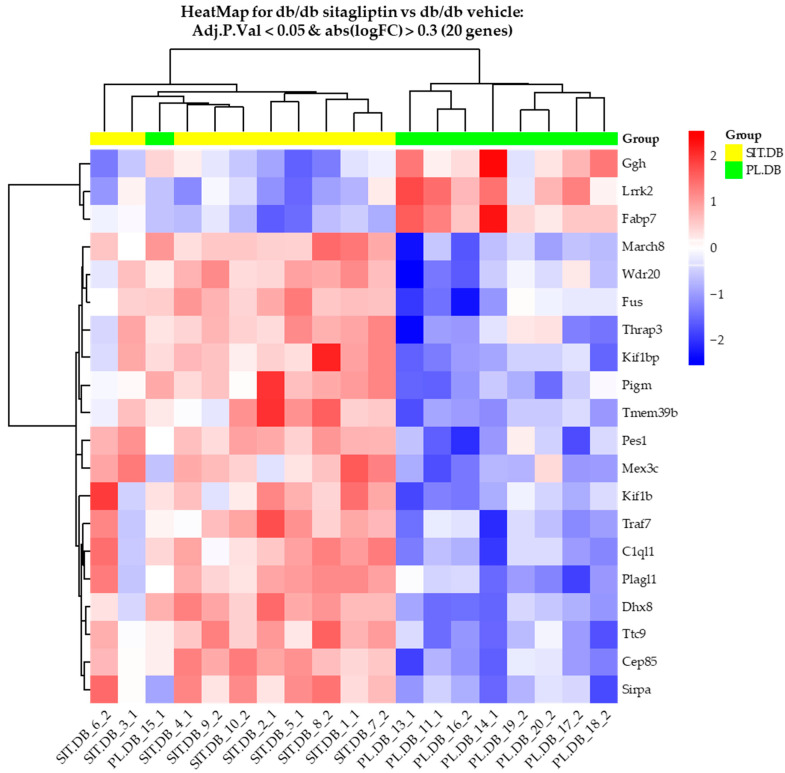
Comparison of retinal expression patterns between sitagliptin-treated and vehicle-treated db/db mice. (**A**) HeatMap for comparison of sitagliptin-treated db/db mice and vehicle-treated db/db mice with genes with an adjusted *p*-value of less than 0.05 and an absLogFC greater than 0.3 (20 genes). High and low row z-scores are represented in red and blue, respectively (*n* = 10).

**Figure 2:**
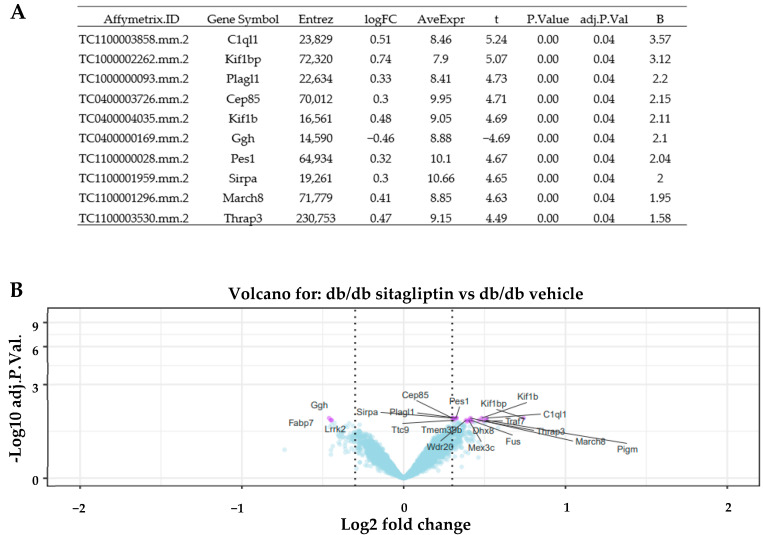
Most differentiated genes. (**A**) Table of top 10 genes more differentially expressed in the comparison between vehicle-treated and sitagliptin-treated mice. AveExpr is the average expression of the gene across all the arrays in log2 scale. t is a “moderated-t” statistic similar to the usual Student’s t statistic (*n* = 10). (**B**) Volcano plot of db/db sitagliptin and db/db vehicle comparison. Genes are shown in purple when adjusted *p*-value was under 0.05, and absolute logarithmic fold change was above 0.3. Gene symbols are shown for the top 20 most significant genes (*n* = 10).

Focusing on the effect of the topical treatment with sitagliptin, we only continued exploring the “db/db sitagliptin vs. db/db vehicle” comparison. HeatMap clustering between both groups using transcripts with an absLogFC ≥ 0.3 and an Adj.P.Val < 0.05 revealed two main clusters of different expression patterns (Figure 1). With the exception of sample “Db/db vehicle_15_1”, all samples of each compared condition were grouped together in the same cluster. Genes more differentially expressed are displayed in a summary table (top 10) and in a volcano plot (top 20) (Figure 2A,B).

### 2.3. Gene Set Enrichment Analyses (GSEA) Revealed That the Most Differentiated Biological Process Is Neurotransmission

**Figure 3:**
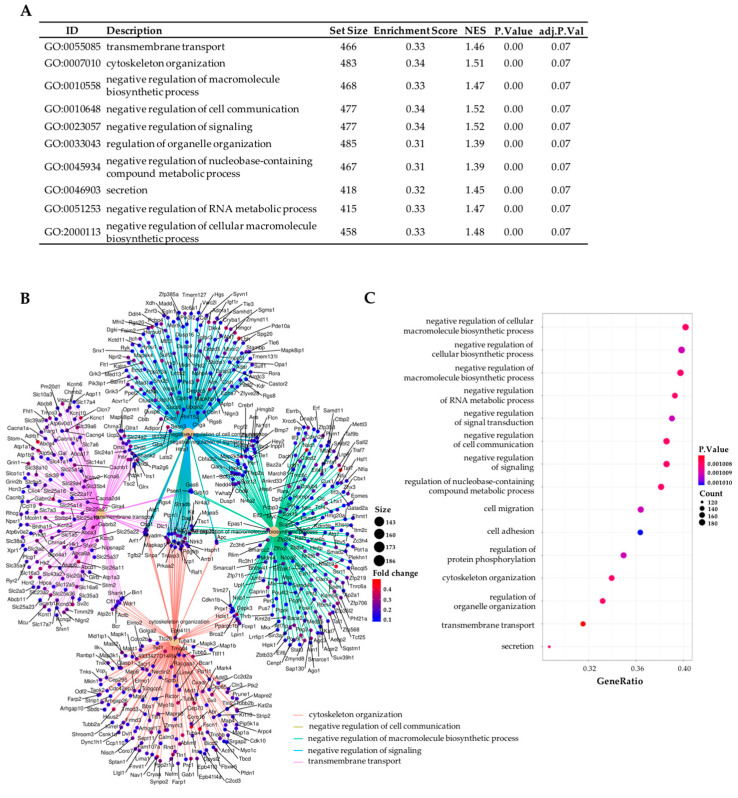
Enrichment analysis of the transcriptome study between vehicle-treated db/db mice and db/db mice treated with sitagliptin using the GO Database (BP subcategory). (**A**) Table of top 10 enriched GO terms (BP) for the comparison “db/db sitagliptin vs. db/db vehicle”. (**B**) Network plot of the top five enriched GO term (BP) for “db/db sitagliptin vs. db/db vehicle”. (**C**) Dot plot of the top 15 enriched GO terms (BP) for the “db/db sitagliptin vs. db/db vehicle” comparison. Results were adjusted with a *p*-value less than 0.25 The results shown correspond to the top enriched terms with an adjusted *p*-value < 0.07.

GSEA is a powerful analysis method for interpreting gene expression data [11]. The analysis of biological significance was approached by GSEA using two different annotation databases: the Go Ontology Database (GO) (Biological Process (BP) subcategory) and the Reactome Pathway Database. Most enriched GO (BP) and Reactome terms revealed neurotransmission to be the most differentiated biological process between the transcriptomes of db/db mice treated with sitagliptin and those of the vehicle-treated mice. In GO analysis, more general and non-cell-type-specific terminology was found: transmembrane transport, cytoskeleton organization, secretion, negative regulation of cell communication, etc. (Figure 3A–C).

**Figure 4:**
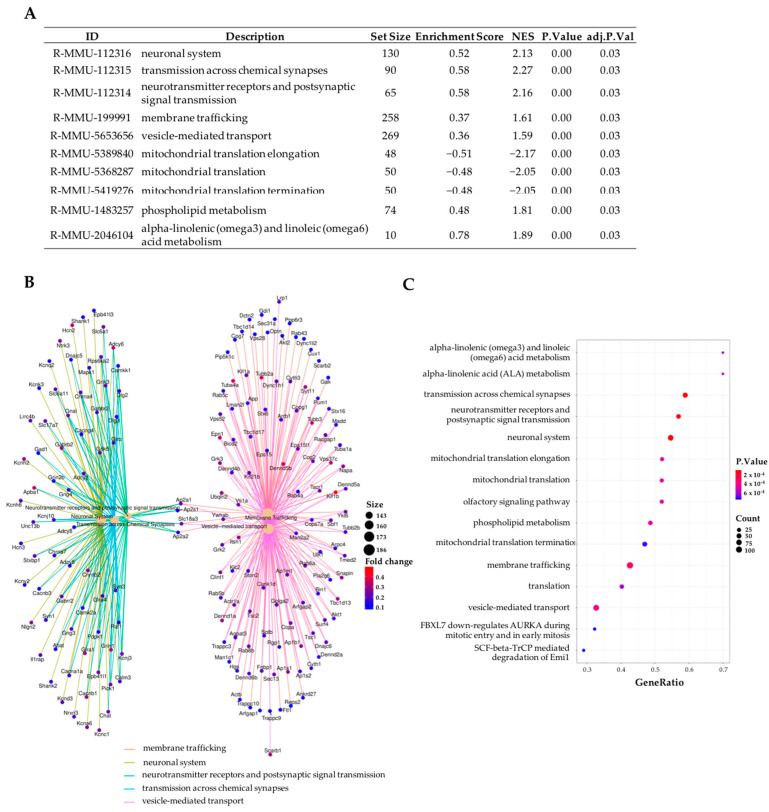
Enrichment analysis of the transcriptome comparison between vehicle-treated db/db mice and db/db mice treated with sitagliptin using the Reactome Pathway Database. (**A**) Table of the top 10 enriched pathways (Reactome Pathway Database) for the comparative study “db/db sitagliptin vs. db/db vehicle”. (**B**) Network plot of the top five enriched pathways for the comparison between db/db sitagliptin and db/db vehicle conditions (Reactome Pathway Database). (**C**) Dot plot of the top 15 enriched pathways (Reactome Pathway Database). The results shown correspond to the top enriched terms with an adjusted *p*-value < 0.05.

**Figure 5:**
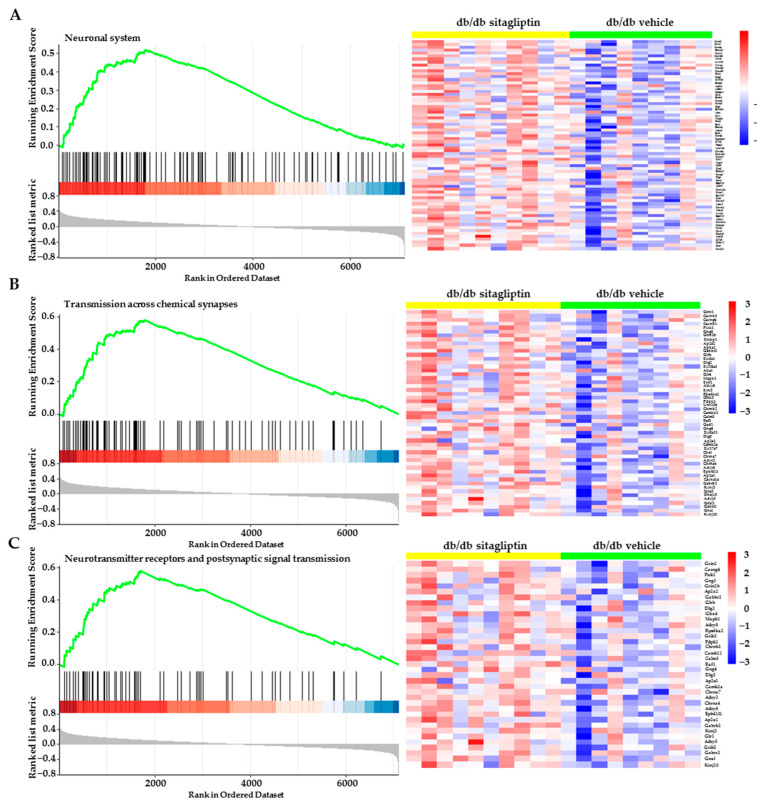
Gene set enrichment analysis (GSEA). (**A**–**C**) Heatmaps of the most enriched neurotransmission-related pathways between vehicle-treated db/db mice and sitagliptin-treated db/db mice.

Nevertheless, Reactome analysis showed us the influence of sitagliptin in the neuronal components of the retina through terms such as: neuronal system, transmission across chemical synapses, neurotransmitters receptors and postsynaptic signal transmission, membrane trafficking, vesicle-mediated transport, etc. (Figure 4A–C). A positive enrichment score or NES indicates that the enriched term is mainly composed of up-regulated genes, whereas a negative score indicates the opposite. In relation to the most enriched biological pathways, the specific heatmaps show clear differential expression patterns between both groups and also expose which genes belong to these pathways (Figure 5A–C).

**Figure 6:**
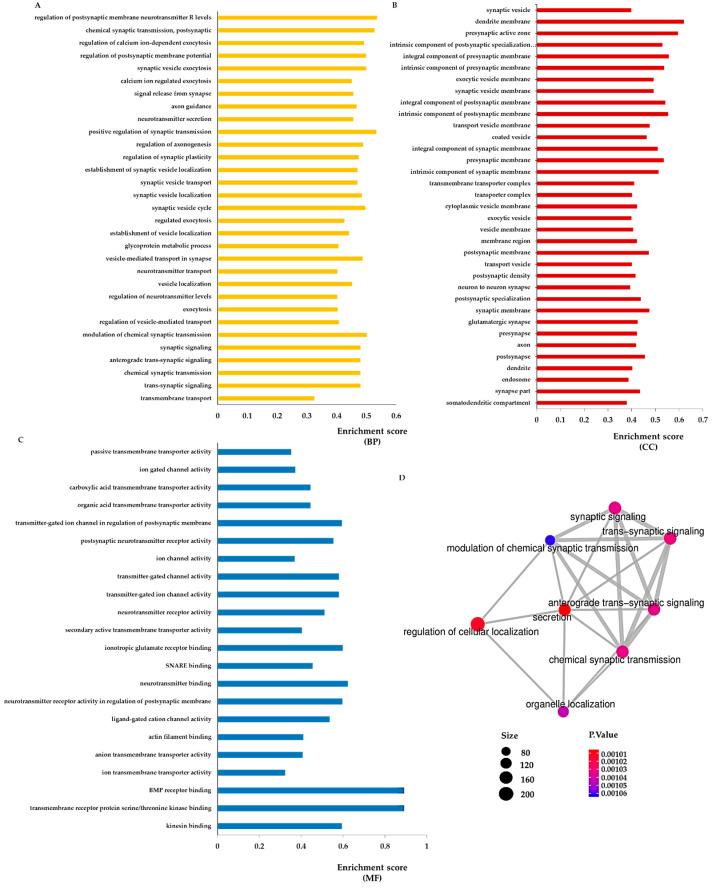
Sitagliptin-enhanced pathways related to synaptic transmission. (**A**–**C**) Most enriched terms involved in neurotransmission between sitagliptin-treated db/db mice and db/db mice treated with vehicle using the GO database (Biological Process (BP), Cellular Component (CC) and Molecular Function (MF) subcategories, respectively) (*p*-value < 0.005). X-axis shows the enrichment score in each category. (**D**) Cluster related to synaptic transmission from the GO enrichment map of the top 60 enriched terms (BP) between db/db sitagliptin and db/db vehicle.

Reanalysis of the GO study, not only using the BP category but also incorporating the Molecular Function (MF) and Cellular Component (CC) categories and limiting the search to neuronal terms, confirmed the Reactome results (Figure 6A–C). An enrichment map of the most differentiated gene sets associated with synaptic transmission (BP category) can also be observed for the sitagliptin vs. vehicle comparison in Figure 6D.

### 2.4. Topical Administration of Sitagliptin Prevented DR-Induced Down-Regulation of Genes Related to Synapse Formation, Maintenance and Synaptic Transmission

**Figure 7:**
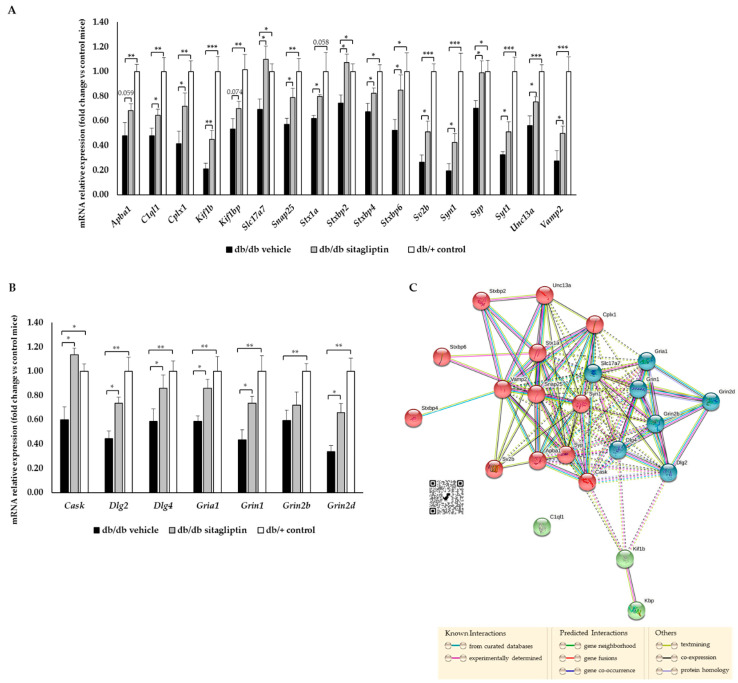
Study of gene expression. (**A**) RT-PCR analysis of genes related to synapse formation and neurotransmission at presynaptic level in db/db mice treated with vehicle (black bars) or sitagliptin eye drops (gray bars) and in non-diabetic mice (white bars) (*n* = 4). * *p* < 0.05, ** *p* < 0.01, *** *p* < 0.001. (**B**) RT-PCR analysis of genes associated with neurotransmission at postsynaptic level in db/db mice treated with vehicle (black bars) or sitagliptin eye drops (gray bars) and in non-diabetic mice (white bars) (*n* = 4). * *p* < 0.05, ** *p* < 0.01. (**C**) Gene interactions of studied genes (STRING ver. 11.5). The red cluster displays genes related to presynaptic proteins, the blue cluster displays those related to postsynaptic proteins and green cluster displays those related to proteins involved in synapse formation. Dashed lines represent interactions between genes from different clusters, while solid lines are the interactions of genes of the same cluster.

Most differentiated genes from the top enriched terms are not the main regulators of these biological processes. In view of this, gene expression of other and more crucial proteins was also addressed. Diabetic mice, in comparison to db/+ control mice, presented a significant down-regulation of several genes related to synapse formation and maintenance (complement C1q-like 1 (*C1ql1*); kinesin family member 1B (*Kif1b*); KIF-1 binding protein (*Kif1bp*)), synaptic transmission at presynaptic level (amyloid beta precursor protein-binding family A member 1 (*Apba1*); complexin 1 (*Cplx1*); solute carrier family 17 member 7 (*Slc17a7*); synaptosome-associated protein 25 (*Snap25*); syntaxin-1A (*Stx1a*); syntaxin-binding protein 2, 4 and 6 (*Stxbp2*, *Stxbp4* and *Stxbp6*); synaptic vesicle glycoprotein 2B (*Sv2b*); synapsin I (*Syn1*); synaptophysin (*Syp*); synaptotagmin (*Syt1*); Unc-13 homolog A (*Unc13A*); vesicle-associated membrane protein 2 (*Vamp2*)) and postsynaptic level (calcium/calmodulin-dependent serine protein kinase (*Cask*); discs large MAGUK scaffold protein 2 and 4 (Dlg2 and Dlg4); glutamate ionotropic receptor AMPA-type subunit 1 (Gria1); glutamate ionotropic receptor NMDA-type subunit 1, 2B and 2D (Grin1, Grin2b and Grin2d)) (Figure 7A,B). Nevertheless, topical administration of sitagliptin in db/db mice significantly prevented these abnormal expression patterns (Figure 7A,B). In addition, STRING interactions between all the analyzed genes demonstrated the close relationship linking all of them (Figure 7C).

## 3. Discussion

In the context of shedding light on the mechanisms behind the beneficial effects that DPP-4i exerted on an experimental model of DR, we provide a preliminary and comparative study of the retinal transcriptomes of db/db mice topically treated with sitagliptin or vehicle. Our results evidence that neurotransmission, wherein sitagliptin has a protective role, is the main biological process affected by DR. A detailed characterization of the genes involved in this improvement is also provided.

Proper functioning of retinal synapses is essential for the neural processing of visual perception. Any morphological change of synaptic terminals or protein content could impair neuronal behaviour and, consequently, the efficiency of the retina to sense and process light stimuli. DR has been widely associated with synaptic loss and with deficits in neurotransmitter release and uptake systems, leading to altered synaptic activity and electrophysiological abnormalities in the retina [12].

Using two different annotations databases (GO and Reactome) and under restrictive statistical conditions, the retinal GSEA revealed positive enrichment after sitagliptin treatment of multiple terms linked to synaptic transmission, such as: neuronal system, transmission across chemical synapses, neurotransmitters receptors and postsynaptic signal transmission, neurotransmitter transport, regulation of postsynaptic membrane potential, membrane trafficking, vesicle-mediated transport in synapse, axon guidance, etc. The neuroprotective role of DPP-4i in relation to eye complications, where synaptic transmission is altered, has been already reported. In a mouse model of retinitis pigmentosa, sitagliptin preserved presynaptic and postsynaptic elements and synaptic contacts between photoreceptors and bipolar/horizontal cells [13]. Furthermore, in type I diabetes STZ rats with continued hyperglycaemia, sitagliptin prevented neuronal cell death in the retina [8,14]. In both studies, sitagliptin was administered orally, and the effects can be attributed to the systemic improvement due to the lowering of blood glucose levels. Nevertheless, our group demonstrated that sitagliptin eye drops are able, in a non-glycaemia-dependent fashion, to significantly prevent the diabetes-induced synaptic failure that occurred in the retina of db/db mice, evidencing the intrinsic neuroprotective properties of DPP-4i [15]. Considering these previous studies, the GSEA results obtained reveal that, among the different beneficial effects of DPP-4i in DR models, retinal neuroprotection is the most relevant one. In addition, it seems that the retinal GLP-1/GLP-1R pathway is the main mediator of these neuroprotective effects [16]. Nonetheless, independent and synergic mechanisms of DPP-4i should not be ruled out [17].

Current evidence does not indicate any beneficial effect of the systemic administration of DDP-4i on DR in addition to their hypoglycemic action. In fact, systematic reviews and meta-analyses reported a neutral effect of DPP-4i on DR [18,19,20]. This can be explained because DPP-4i, and, in particular, sitagliptin, is unable to cross the blood–retinal barrier [21,22]. For this reason, we examined the effect of topical administration of sitagliptin. By using this route, sitagliptin was able to reach the retina and exert its effects independently from its capacity to lower blood glucose levels. In fact, this is an advantage because it permitted us to demonstrate the direct effect of the drug in the retina independently of metabolic control. It should be noted that the capacity of antidiabetic agents to lower blood glucose levels is a confounding factor in studies aimed at evaluating their potential effect in the development or progression of DR.

It is not entirely possible to elucidate the neuroprotective impact that dependent or independent GLP-1/GLP-1R pathways have; however, we provide a characterization of some potential targets of both mechanisms (direct or indirect targets). On the one hand, *C1ql1*, the most differentiated gene, codifies for a secreted protein that interacts with hippocampal receptors that promote spinogenesis, synaptogenesis and synaptic territory constitution, essential processes for a functional neuronal connectivity [23]. On the other hand, *Kif1bp* and *Kif1b* genes (top two and five) give rise to a KIF1B-KBP complex, necessary for proper axon elongation [24]. The presence of three genes intimately linked to crucial functions of the neuronal system among the five most differentiated genes is a clear proof of the neuroprotective role of sitagliptin that cannot go unnoticed.

In addition, multiple genes involved in neurotransmitter uptake and release are up-regulated by sitagliptin (*Apba1*, *Cplx1*, *Slc17a7, Stxbp2*, *Stxbp4*, *Stxbp6*, *Sv2b* and *Unc13a*). The proteins that these genes codify are responsible for vesicle biogenesis, mobilization, docking, fusion and recycling [25]. Nevertheless, they are not the most characterized proteins of this biological process, and, therefore, in our RT-PCR validation, we also assessed the mRNA levels of *Snap25*, *Stx1a*, *Syn1*, *Syp*, *Syt1* and *Vamp2*. The core of the neurotransmitter release machinery is composed of the N-ethylmaleimide-sensitive factor attachment protein (SNAP) receptors (SNAREs) syntaxin-1 (*Stx1a*), SNAP-25 (*Snap25*) and synaptobrevin (*Vamp2*), which form the SNARE complex, a four-helix bundle that brings close both vesicle and plasma membrane for proper vesicle docking and fusion. Munc-18 (*Stxbp2*, *Stxbp4*, *Stxbp6*) and Munc-13 (*Unc13a*) modulate SNARE complex formation. Munc-18 binds to a “closed” conformation of syntaxin-1 and to synaptobrevin to stabilize the assembly of the SNARE complex. Munc-13 is responsible for syntaxin-1 “opening” and permits vesicle binding to the plasma membrane [26]. Mint-1 (*Apba1*) mediates the function of Munc-18 [27]. Synaptotagmin 1 (*Syt1*) is a transmembrane protein present in synaptic vesicles which senses and couples Ca^2+^ influx to synchronize neurotransmitter releases. The role of complexin I (*Cplx1*) is not well understood, but it has been related to vesicle fusion [28]. Synaptophysin (*Syp*) is the most abundant synaptic protein, and it modulates the endocytosis of synaptic vesicles [29]. Synapsin I (*Syn1*) orchestrates the reserve pool of synaptic vesicles available for exocytosis and organises the abundance of vesicles at presynaptic terminals [30,31]. Synaptic vesicle glycoprotein 2B (SV2B) (*Sv2b*) stabilizes the vesicle content, maintains and orients the releasable pool of vesicles and modulates vesicular calcium sensitivity to coordinate efficient neurotransmitter release [32]. Finally, vesicular glutamate transporter 1 (VGLUT1) (*Slc17a7*) refills presynaptic recycling vesicles with glutamate, the major excitatory transmitter in the retina, the excitotoxicity of which is a hallmark of DR [33].

Not only genes related to neurotransmitter uptake and release but also genes linked to the next biological step, the postsynaptic processing, have been found. In our transcriptomic analysis, we detected that sitagliptin up-regulates genes that codify for three different subunits of the glutamate ionotropic receptor (N-methyl-D-aspartate) NMDA (*Grin1*, *Grin2b*, *Grin2d*), one subunit of the glutamate α-amino-3-hydroxy-5-methyl-4-isoxazolepropionic acid (AMPA) receptor (*Gria1*), two different postsynaptic density proteins (PSD) (*Dlg2*, *Dlg4*) and the calcium/calmodulin-dependent serine protein kinase (CASK) (*Cask*). Glutamate interacts with both ionotropic and metabotropic receptors to activate and mediate postsynaptic responses. AMPA and NMDA receptors are crucial modulators of synaptic plasticity, the dysregulation of which leads to neurodegenerative processes, including DR [34,35]. PSD-93, PSD-95 and CASK, codified by *Dlg2*, *Dlg4* and *Cask*, respectively, are postsynaptic scaffolding proteins that regulate the synaptic localization of many receptors, channels and signalling proteins [36,37].

In the RT-PCR assay, we found that db/db mice treated with the vehicle, in comparison to non-diabetic animals, presented a down-regulation of all the studied proteins involved in synapse formation, maintenance, neurotransmitter release and postsynaptic response. Sitagliptin eye drops preserved the levels of all these proteins, which reflects its beneficial and significant impact on the retinal neuronal system. As a proper content of synaptic proteins is necessary for the functionality of the retina, we can assume that the results obtained in the present study represent the underlying mechanisms behind the functional recovery (ERG) that we previously reported [8].

It is important to notice the several enriched pathways from our transcriptomic analysis that demonstrate the multiple beneficial effects of DPP-4i not only related to neuroprotection, but also associated with anti-inflammatory or anti-angiogenic properties. Further analysis specifically addressed to confirm this issue is needed.

In summary, topical administration of sitagliptin had neuroprotective effects in an experimental model of DR by preventing the dysregulation of pre- and postsynaptic proteins and other molecules involved in synapse formation and maintenance. This evidence could represent an important approach for the great unmet medical need that diabetic retinopathy represents and even for other retinal diseases in which neurodegeneration/synaptic abnormalities play a key role.

## 4. Materials and Methods

### 4.1. Mice

A total of 24 diabetic male db/db (BKS.Cg-Dock7m +/+ Leprdb/J) mice and 12 non-diabetic mice (db/+; (BKS.Cg-Dock7m + Leprdb/+)) aged 7 weeks were obtained from Charles River Laboratories Inc. (Calco, Italy) for the study. Db/db mice carry a mutated leptin receptor that leads to obesity-induced type 2 diabetes. Animals were bred and maintained in VHIR’s animal facility. Animals had free access to ad libitum food (ENVIGO Global Diet Complete Feed for Rodents, Mucedola, Milan, Italy) and filtered water. In order to minimize variability, animals were randomly housed (block randomization) in groups of 2 mice per cage in Tecniplast GM-500 cages (36 × 19 × 13.5 cm) under standard laboratory conditions at 22 ± 2 °C, with a 12 h light/dark cycle and relative humidity of 50–60%. Each cage held absorbent bedding and nesting material (BioFresh Performance Bedding 1/800 Pelleted Cellulose, Absorption Corp, Ferndale, WA, USA). Blood glucose levels were measured weekly through tail vein sampling (glucose assay kit; Abbott, IL, USA).

All accomplished experiments with animals were adjusted in compliance with European Community (86/609/CEE) and ARVO (Association for Research in Vision and Ophthalmology) tenets for the use of laboratory animals. This study was approved by the Animal Care and Use Committee of Vall d’Hebron Research Institute (VHIR) (CEEA 75/15).

### 4.2. Interventional Study

At the age of 10 weeks, sitagliptin (sitagliptin phosphate monohydrate (Y0001812, Merck KGaA, Darmstadt, Germany)) eyedrops (10 mg/mL; *n* = 12) and vehicle (phosphate-buffered saline (PBS)) eyedrops (*n* = 12) were randomly administered twice per day directly onto the superior corneal surface of each eye of diabetic mice with the aid of a micropipette (5 µL). On day 15, animals (12 weeks of age) received one drop of sitagliptin or vehicle 1 h before euthanasia. Twelve non-diabetic mice, matched by age, were used as a control group.

### 4.3. Retinal Tissue Processing

On day 15, each animal received a 200 µL intraperitoneal injection of anesthesia prepared with a mix containing 1 mL ketamine (GmbH, Hameln, Germany) and 0.3 mL xylazine (Laboratorios Calier S.A., Barcelona, Spain). Eyes were rapidly enucleated, and the neuroretinas were dissected. The neuroretina of each animal was directly stored in an empty tube at −80 °C for transcriptomic experiments. The neuroretinas were introduced in individual tubes with 140 µL of TRIzol reagent (15596018, Invitrogen^TM^, Carlsbad, CA, USA) until RNA extraction. For the RNA extraction, neuroretinas were treated with DNase (18068015, ThermoFisher Scientific, Waltham, MA, USA) to remove genomic contamination and were purified on RNeasy MinElute column (74106, Qiagen, Hilden, Germany). After supernatant elimination, RNA sediment was obtained and resuspended in 30 µL of RNAse free water (AM9937, ThermoFisher Scientific, Waltham, MA, USA). An Agilent 2100 Bioanalyzer and a NanoDrop Spectrophotometer were used for sample integrity and quantity, respectively.

### 4.4. Transcriptome Analysis

The data for the analysis were obtained from Genomic’s UAT core facility at VHIR, where the microarrays were performed. The study was based on 30 samples (the 10 highest quality RNA samples of each group) hybridized in Clariom S Mouse Arrays (ThermoFisher Scientific, Waltham, MA, USA). Bioinformatics analysis was carried out in the Statistics and Bioinformatics Unit (UEB) at VHIR. Quality approaches (principal component analysis (PCA), hierarchical clustering and heatmaps depicting distances between arrays) were performed before normalization. Any sample was excluded. All samples were normalized following the robust microchip average (RMA) algorithm [38,39]. PCA and hierarchical clustering quality controls were assessed again, and one outlier from the vehicle group was excluded. Using principal variance component analysis (PVCA), we estimated that the main cause of variance was the batch effect. The analysis to select differentially expressed genes (DEG) was based on adjusting a linear model with empirical Bayes moderation of the variance, including a batch factor to adjust batch effects. This is a technique similar to ANOVA specifically developed for microarray data analysis by Gordon K Smyth [40]. *p*-values were adjusted to obtain strong control over the false discovery rate using the Benjamini and Hochberg method. Statistical significance was set at 0.05 for adjusted *p*-values. The analysis of biological significance was based on gene set enrichment analysis (GSEA) using clusterProfiler package in R/Bioconductor [41], which implements the GSEA algorithm proposed by Subramanian [11]. In GSEA, the genes can be ordered in a ranked list according to their differential expression between the classes using different annotation databases (Gene Ontology (GO) and Reactome Pathway Knowledge base). The statistical analysis was performed using the statistical language “R” (R, version 3.5.1 (2 July 2018), copyright (C) 2018, The R Foundation for Statistical Computing) and the libraries developed for the microarray analysis in the Bioconductor Project (www.bioconductor.org) (accessed on 18 October 2021). Functional association networks were constructed and annotated using STRING version 11.5 (https://string-db.org/) (accessed on 18 October 2021). The *Mus musculus* genome was used as background genome. The data discussed in this publication were deposited in NCBI’s Gene Expression Omnibus (Edgar et al., 2002) and are accessible through GEO Series accession number GSE219084 (https://www.ncbi.nlm.nih.gov/geo/query/acc.cgi?acc=GSE219084).

### 4.5. cDNA Reverse Transcription and Quantitative Reverse Transcription Polymerase Chain Reaction (RT-PCR) Assay

According to the manufacturer’s instructions, cDNA reverse transcription was performed in a T100 Thermal Cycler (Bio-Rad, Hercules, CA, USA) using a High-Capacity cDNA Reverse Transcription Kit (4368814, ThermoFisher Scientific, Waltham, MA, USA) and Oligo(dT)18 Primer (SO131, ThermoFisher Scientific, Waltham, MA, USA). RT-PCR was carried out using SYBR Green PCR Master Mix (4309155, Applied Biosystems, Warrington, UK) and a 7.900 HT Sequence Detection System in 384-well optical plates with specific primers (displayed in Table 2). Relative quantities were calculated using the ABI SDS 2.4 RQ software and presented as a ratio between them and the endogenous controls (*B2m* and *Actin*).

### 4.6. Statistical Analysis of RT-PCR

Data are presented as mean ± SEM. Statistical evaluations were performed with Student’s unpaired *t*-test for RT-PCR. When multiple comparisons were performed, one-way ANOVA followed by the Bonferroni test was used. Statistical significance was set at *p* < 0.05.

## 5. Conclusions

This work indicates that the prevention of synaptic abnormalities plays a key role in the reported beneficial effects of topical (eye drops) administration of sitagliptin. These results, in addition to its already demonstrated ability to reduce inflammation, glial activation and vascular leakage, suggest that topical administration of sitagliptin may become a new therapeutic strategy against early stages of DR.

## Figures and Tables

**Table 1:** Multiple comparisons of DEGs between groups. Summary table of the number of DEGs acquired for each comparison at distinct significance thresholds (green: db/db sitagliptin vs. db/+ control; red: db/db vehicle vs. db/+ control; blue: db/db sitagliptin vs. db/db vehicle) (*n* = 10).

	db/db Sitagliptin vs. db/+ Control	db/db Vehicle vs. db/+ Control	db/db Sitagliptin vs. db/db Vehicle
UpReg_B	65	15	33
DownReg_B	20	13	9
UpRegAdj0.01	18	5	0
DownRegAdj0.01	6	3	0
UpRegAdj0.05	108	10	17
DownRegAdj0.05	33	9	3
UpRegAdj0.15	369	16	185
DownRegAdj0.15	122	14	65
UpRegAdj0.25	728	24	494
DownRegAdj0.25	291	19	195
UpRegP0.01	363	71	275
DownRegP0.01	116	56	96
UpRegP0.05	852	218	82
DownRegP0.01	376	223	329

**Table 2:** Primers used for RT-PCR.

Primers		Nucleotide Sequence
*Actb*	Forward (5′-3′)	5′-CTAAGGCCAACCGTGAAAG -3′
	Reverse (5′-3′)	5′-CAGTATGTTCGGCTTCCCATTC-3′
*Apba1*	Forward (5′-3′)	5′-GGTGCTGAGTCATCAAGCATAC-3′
	Reverse (5′-3′)	5′-GAACTTCAACGTAGGTTGGGAA-3′
*B2m*	Forward (5′-3′)	5′-GTATGCTATCCAGAAAACCC-3′
	Reverse (5′-3′)	5′-CTGAAGGACATATCTGACATC-3′
*C1ql1*	Forward (5′-3′)	5′-GGCACCTACTTTTTCACCTACC-3′
	Reverse (5′-3′)	5′-AGTCGTAGTTCTGGTCTGCAT-3′
*Cask1*	Forward (5′-3′)	5′-TGAAGAAGTAGTCAAACTGCCAG-3′
	Reverse (5′-3′)	5′-TTTGTCCCGTACATTGCATCC-3′
*Cplx1*	Forward (5′-3′)	5′-AGTTCGTGATGAAACAAGCCC-3′
	Reverse (5′-3′)	5′-TCTTCCTCCTTCTTAGCAGCA-3′
*Dlg2*	Forward (5′-3′)	5′-CTGTCACGAGGCAGGAAATAAA-3′
	Reverse (5′-3′)	5′-CGACTTCGTAGTCACGCTTTG-3′
*Dlg4*	Forward (5′-3′)	5′-TGAGATCAGTCATAGCAGCTACT-3′
	Reverse (5′-3′)	5′-CTTCCTCCCCTAGCAGGTCC-3′
*Gria1*	Forward (5′-3′)	5′-AAAGGAGTGTACGCCATCTTTG-3′
	Reverse (5′-3′)	5′-TGTCAACGGGAAAACTTGGAG-3′
*Grin1*	Forward (5′-3′)	5′-ATGCACCTGCTGACATTCG-3′
	Reverse (5′-3′)	5′-TATTGGCCTGGTTTACTGCCT-3′
*Grin2b*	Forward (5′-3′)	5′-GCCATGAACGAGACTGACCC-3′
	Reverse (5′-3′)	5′-GCTTCCTGGTCCGTGTCATC-3′
*Grin2d*	Forward (5′-3′)	5′-GCTGCGAGACTATGGCTTCC-3′
	Reverse (5′-3′)	5′-CCAGTGACGGGTTTACCAGAAA-3′
*Kif1b*	Forward (5′-3′)	5′-GTCAATCGAATGAACGACCTGG-3′
	Reverse (5′-3′)	5′-GCCGATGCAAAAAGTTGAACTG-3′
*Kif1bp*	Forward (5′-3′)	5′-TCTTGACCCGACTGAGCATTT-3′
	Reverse (5′-3′)	5′-ATAATGAGCGGCCTTCTCGAA-3′
*Slc17a7*	Forward (5′-3′)	5′-GGTGGAGGGGGTCACATAC-3′
	Reverse (5′-3′)	5′-AGATCCCGAAGCTGCCATAGA-3′
*Snap25*	Forward (5′-3′)	5′-CAACTGGAACGCATTGAGGAA-3′
	Reverse (5′-3′)	5′- GGCCACTACTCCATCCTGATTAT-3′
*Stx1a*	Forward (5′-3′)	5′-CGCTGTCCCGAAAGTTTGTG-3′
	Reverse (5′-3′)	5′-GTGTCTGGTCTCGATCTCACT-3′
*Stxbp2*	Forward (5′-3′)	5′-AAGGCGGTGGTAGGGGAAA-3′
	Reverse (5′-3′)	5′-CAACAGGATGACAAGATTCGCA-3′
*Stxbp4*	Forward (5′-3′)	5′-ACAGGTCTAGGTCTGAAGATCC-3′
	Reverse (5′-3′)	5′-CATCCTTGTAACAGTCACCTCC-3′
*Stxbp6*	Forward (5′-3′)	5′-CTCTTGATGAAAGAATGCTGGGA-3′
	Reverse (5′-3′)	5′-TGACCTTCGTGATAGATGCCT-3′
*Sv2b*	Forward (5′-3′)	5′-AGGTATCGGGACAACTATGAGG-3′
	Reverse (5′-3′)	5′-GCCTTCTGTAACATCGCTCTGT-3′
*Syn1*	Forward (5′-3′)	5′-AATCACAAAGAGATGCTCAG-3′
	Reverse (5′-3′)	5′-GGACACGCACATCATATTTAG-3′
*Syp*	Forward (5′-3′)	5′-TGCCAACAAGACGGAGAGTG-3′
	Reverse (5′-3′)	5′-TAGTGCCCCCTTTAACGCAG-3′
*Syt1*	Forward (5′-3′)	5′-ACCCTGGGCTCTGTATCCC-3′
	Reverse (5′-3′)	5′- CCCTGACCACTGAGTGCAAA-3′
*Unc13A*	Forward (5′-3′)	5′-GCTGTGCGTGGGAGTCAAA-3′
	Reverse (5′-3′)	5′-CAGCTATGGTAGTGCTCTTCA-3′
*Vamp2*	Forward (5′-3′)	5′-ATCATCGTTTACTTCAGCAC-3′
	Reverse (5′-3′)	5′-TGAAAGATATGGCTGAGAGG-3′

## Data Availability

The raw data of this manuscript are in the GEO repository. Accession “GSE219084” is currently private and is scheduled to be released on 30 November 2025. When the manuscript is published, it will be changed to open access.

## References

[1] Leasher J.L., Bourne R.R., Flaxman S.R., Jonas J.B., Keeffe J., Naidoo K., Pesudovs K., Price H., White R.A., Wong T.Y. (2016). Vision Loss Expert Group of the Global Burden of Disease Study. Global estimates on the number of people blind or visually impaired by diabetic retinopathy: A meta-analysis from 1990 to 2010. Diabetes Care.

[2] Wong T.Y., Cheung C.M., Larsen M., Sharma S., Simó R. (2016). Diabetic retinopathy. Nat. Rev. Dis. Primers.

[3] Simó R., Hernández C. (2015). Novel approaches for treating diabetic retinopathy based on recent pathogenic evidence. Prog. Retin. Eye Res..

[4] Solomon S.D., Chew E., Duh E.J., Sobrin L., Sun J.K., VanderBeek B.L., Wykoff C.C., Gardner T.W. (2017). Diabetic Retinopathy: A Position Statement by the American Diabetes Association. Diabetes Care.

[5] Simó R., Stitt A.W., Gardner T.W. (2018). Neurodegeneration in diabetic retinopathy: Does it really matter?. Diabetologia.

[6] Simó R., Simó-Servat O., Bogdanov P., Hernández C. (2021). Neurovascular Unit: A New Target for Treating Early Stages of Diabetic Retinopathy. Pharmaceutics.

[7] Salcedo I., Tweedie D., Li Y., Greig N.H. (2012). Neuroprotective and neurotrophic actions of glucagon-like peptide-1: An emerging opportunity to treat neurodegenerative and cerebrovascular disorders. Br. J. Pharmacol..

[8] Hernández C., Bogdanov P., Solà-Adell C., Sampedro J., Valeri M., Genís X., Simó-Servat O., García-Ramírez M., Simó R. (2017). Topical administration of DPP-IV inhibitors prevents retinal neurodegeneration in experimental diabetes. Diabetologia.

[9] Hernández C., Bogdanov P., Corraliza L., García-Ramírez M., Solà-Adell C., Arranz J.A., Arroba A.I., Valverde A.M., Simó R. (2016). Topical administration of GLP-1 receptor agonists prevents retinal neurodegeneration in experimental diabetes. Diabetes.

[10] Röhrborn D., Wronkowitz N., Eckel J. (2015). DPP4 in Diabetes. Front. Immunol..

[11] Subramanian A., Tamayo P., Mootha V.K., Mukherjee S., Ebert B.L., Gillette M.A., Paulovich A., Pomeroy S.L., Golub T.R., Lander E.S. (2005). Gene set enrichment analysis: A knowledge-based approach for interpreting genome-wide expression profiles. Proc. Natl. Acad. Sci. USA.

[12] Barber A.J., Baccouche B. (2017). Neurodegeneration in diabetic retinopathy: Potential for novel therapies. Vision. Res..

[13] Kutsyr O., Arango-Gonzalez B., Fernandez-Sanchez L., Maneu V., Lax P., Ambrosio A.F., Ueffing M., Cuenca N. (2019). Dipeptidyl deptidase-IV inhibition by sitagliptin slows down retinal neurodegeneration in rd10 mice retinas. Investig. Ophthalmol. Vis. Sci..

[14] Gonçalves A., Marques C., Leal E., Ribeiro C.F., Reis F., Ambrósio A.F., Fernandes R. (2014). Dipeptidyl peptidase-IV inhibition prevents blood-retinal barrier breakdown, inflammation and neuronal cell death in the retina of type 1 diabetic rats. Biochim. Biophys. Acta.

[15] Ramos H., Bogdanov P., Sabater D., Huerta J., Valeri M., Hernández C., Simó R. (2021). Neuromodulation Induced by Sitagliptin: A New Strategy for Treating Diabetic Retinopathy. Biomedicines.

[16] Zhang Y., Liu Y., Xu J., Sun Q., Yu F., Cheng J., Peng B., Liu W., Xiao Z., Yin J. (2018). Inhibition of DPP4 enhances inhibitory synaptic transmission through activating the GLP-1/GLP-1R signaling pathway in a rat model of febrile seizures. Biochem. Pharmacol..

[17] Dietrich N., Kolibabka M., Busch S., Bugert P., Kaiser U., Lin J., Fleming T., Morcos M., Klein T., Schlotterer A. (2016). The DPP4 inhibitor linagliptin protects from experimental diabetic retinopathy. PLoS ONE.

[18] Chung Y.R., Ha K.H., Kim H.C., Park S.J., Lee K., Kim D.J. (2019). Dipeptidyl Peptidase-4 Inhibitors versus Other Antidiabetic Drugs Added to Metformin Monotherapy in Diabetic Retinopathy Progression: A Real World-BasedCohort Study. Diabetes Metab. J..

[19] Tang H., Li G., Zhao Y., Wang F., Gower E.W., Shi L., Wang T. (2018). Comparisons of diabetic retinopathy events associated with glucose-lowering drugs in patients with type 2 diabetes mellitus: A network meta-analysis. Diabetes Obes. Metab..

[20] Taylor O.M., Lam C. (2020). The Effect of Dipeptidyl Peptidase-4 Inhibitors on Macrovascular and Microvascular Complications of Diabetes Mellitus: A Systematic Review. Curr. Ther. Res. Clin. Exp..

[21] Fura A., Khanna A., Vyas V., Koplowitz B., Chang S.Y., Caporuscio C., Boulton D.W., Christopher L.J., Chadwick K.D., Hamann L.G. (2009). Pharmacokinetics of the dipeptidyl peptidase 4 inhibitor saxagliptin in rats, dogs, and monkeys and clinical projections. Drug Metab. Dispos..

[22] Fuchs H., Binder R., Greischel A. (2009). Tissue distribution of the novel DPP-4 inhibitor BI 1356 is dominated by saturable binding to its target in rats. Biopharm. Drug Dispos..

[23] Sigoillot S.M., Iyer K., Binda F., González-Calvo I., Talleur M., Vodjdani G., Isope P., Selimi F. (2015). The Secreted Protein C1QL1 and Its Receptor BAI3 Control the Synaptic Connectivity of Excitatory Inputs Converging on Cerebellar Purkinje Cells. Cell Rep..

[24] Drerup C.M., Lusk S., Nechiporuk A. (2016). Kif1B Interacts with KBP to Promote Axon Elongation by Localizing a Microtubule Regulator to Growth Cones. J. Neurosci..

[25] Becherer U., Rettig J. (2006). Vesicle pools, docking, priming, and release. Cell Tissue Res..

[26] Stepien K.P., Prinslow E.A., Rizo J. (2019). Munc18-1 is crucial to overcome the inhibition of synaptic vesicle fusion by αSNAP. Nat. Commun..

[27] Okamoto M., Südhof T.C. (1997). Mints, Munc18-interacting proteins in synaptic vesicle exocytosis. J. Biol. Chem..

[28] Courtney N.A., Bao H., Briguglio J.S., Chapman E.R. (2019). Synaptotagmin 1 clamps synaptic vesicle fusion in mammalian neurons independent of complexin. Nat. Commun..

[29] Kwon S.E., Chapman E.R. (2011). Synaptophysin regulates the kinetics of synaptic vesicle endocytosis in central neurons. Neuron.

[30] Bähler M., Benfenati F., Valtorta F., Greengard P. (1990). The synapsins and the regulation of synaptic function. Bioessays.

[31] Bykhovskaia M. (2011). Synapsin regulation of vesicle organization and functional pools. Semin. Cell Dev. Biol..

[32] Stout K.A., Dunn A.R., Hoffman C., Miller G.W. (2019). The Synaptic Vesicle Glycoprotein 2: Structure, Function, and Disease Relevance. ACS Chem. Neurosci..

[33] Martineau M., Guzman R.E., Fahlke C., Klingauf J. (2017). VGLUT1 functions as a glutamate/proton exchanger with chloride channel activity in hippocampal glutamatergic synapses. Nat. Commun..

[34] Huang S., Chen L., Bladen C., Stys P.K., Zamponi G.W. (2018). Differential modulation of NMDA and AMPA receptors by cellular prion protein and copper ions. Mol. Brain.

[35] Smith S.B. (2002). Diabetic Retinopathy and the NMDA Receptor. Drug News Perspect.

[36] Jeong J., Pandey S., Li Y., Badger J.D., Lu W., Roche K.W. (2019). PSD-95 binding dynamically regulates NLGN1 trafficking and function. Proc. Natl. Acad. Sci. USA.

[37] Hsueh Y.P. (2006). The role of the MAGUK protein CASK in neural development and synaptic function. Curr. Med. Chem..

[38] Gentleman R., Carey V., Huber W., Irizarry R.A., Dudoit S. (2005). Bioinformatics and Computational Biology Solutions Using R and Bioconductor.

[39] Irizarry R.A., Hobbs B., Collin F., Beazer-Barclay Y.D., Antonellis K.J., Scherf U., Speed T.P. (2003). Exploration, normalization, and summaries of high density oligonucleotide array probe level data. Biostatistics.

[40] Smyth G.K. (2004). Linear models and empirical bayes methods for assessing differential expression in microarray experiments. Stat. Appl. Genet Mol. Biol..

[41] Wu T., Hu E., Xu S., Chen M., Guo P., Dai Z., Feng T., Zhou L., Tang W., Zhan L. (2021). clusterProfiler 4.0: A universal enrichment tool for interpreting omics data. Innovation.

